# Use of Computer Simulation in Dental Training with Special Reference to Simodont

**DOI:** 10.3390/dj9110125

**Published:** 2021-10-21

**Authors:** Angie Lok-Sze Leung, Conson Yeung, Samantha Chu, Amy Wai-Yee Wong, Ollie Yiru Yu, Chun-Hung Chu

**Affiliations:** Faculty of Dentistry, The University of Hong Kong, Prince Philip Dental Hospital, 34 Hospital Road, Sai Ying Pun, Hong Kong, China; angie667@connect.hku.hk (A.L.-S.L.); conson@hku.hk (C.Y.); chus@connect.hku.hk (S.C.); drawong@hku.hk (A.W.-Y.W.); ollieyu@hku.hk (O.Y.Y.)

**Keywords:** virtual reality, haptics, simulation, education, Simodont

## Abstract

Simulation-based dental education has been increasingly implemented in dental training. Virtual reality simulators are being explored as an adjunct to dental education. Simulation-based dental education could serve as a powerful aid to preclinical instruction. This article provides an overview of how dental simulators can be used in dental instruction and manual dexterity training, utilizing the Simodont dental trainer as a reference. The Simodont dental trainer provides a platform for students to hone their manual dexterity skills and practice repeatedly prior to conventional clinical simulations. Additionally, it can reduce resource wastage. However, the financial cost of setting up and maintaining the system can be high. The high cost would ultimately limit the number of devices each individual school could afford, as a potential drawback to meeting the training needs of many dental students at one time. The machine’s force-feedback mechanism provides trainees with the tactile experience of drilling into various tissues. Students are empowered via self-learning and assessment, with guidance provided for diagnosis and treatment. From training students on basic operative skills to providing basic aptitude tests for entrance examinations, the Simodont dental trainer’s functions and potential for further development may make it a valuable tool in the field of simulation-based dental education.

## 1. Introduction

With the advancement of technology, simulation and virtual reality have facilitated teaching, learning and professional training in dentistry [1]. In disciplines such as dentistry involving unpredictable situations and requiring a certain level of manual dexterity, much attention has been paid to simulation and virtual reality as possible tools with which to provide a realistic training environment for students to practice high-risk procedures. In medicine, medical technologies have already been developed for surgical training, including endoscopy, laparoscopic and neurosurgery simulators. A report on sigmoidoscopy simulator training found that it improved examination times and hand-to-eye skill measures [2].

The acquisition of psychomotor skills makes up a substantial part of the dental curriculum. As dentists perform detailed work on a small scale, hand–eye coordination is crucial for carrying out operative procedures. Restorative dentistry students should gain sufficient hands-on practice in a simulation environment to prevent mishaps caused by human error before they enter the clinical setting [3]. Therefore, simulation-based dental education is being explored as a clinical environment simulator in which preclinical training can be carried out safely.

**Figure 1:**
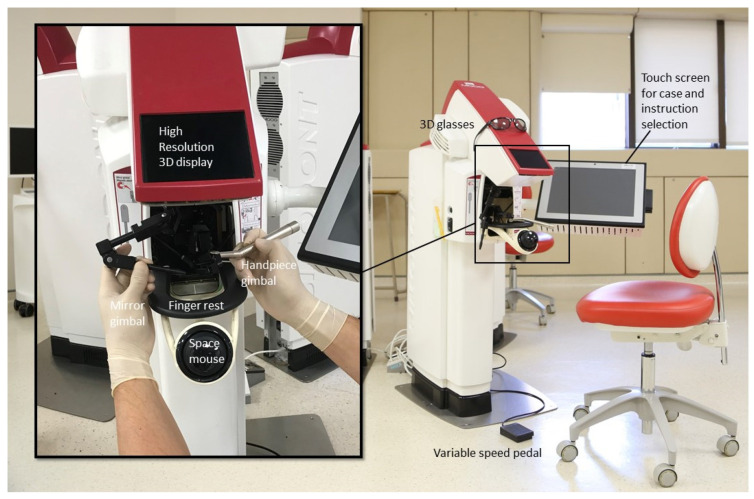
Components of Simodont.

Conventional preclinical training involves mannequin heads mounted on a metal rod. These phantom heads contain a set of plastic maxillary and mandibular teeth. Dental simulation systems are a newer practice method. Example of these include PerioSim, the Simodont dental trainer and DentSim. These simulators utilize computer-assisted simulation, which connects computer learning software to a simulator. The simulator generates 3D images for students to operate on using dental handpieces. The results are sent to the computer for evaluation and feedback [4]. Students can practice on these devices before practicing on phantom heads and, eventually, on real patients. The Simodont dental trainer (MOOG, Nieuw Vennep, The Netherlands) is one such simulator. The Simodont brand is developed by Nissin Dental Products Europe BV (Nieuw-Vennep, The Netherlands). The various components of Simodont Dental Trainer are shown in Figure 1.

Simodont provides a 3D display of the instrument and tooth on-screen in true size [5]. The gimbals on either side of the machine appear in full size on-screen, and one of them functions as a handpiece with which to work on the selected model. The required instruments are selected before starting. A force-feedback mechanism is applied so that the tactile sensation of drilling into the tooth is transmitted when the handpiece touches the model. Auditory and visual effects also are provided for realism [5]. Afterward, the computer evaluates the operation and displays the results on the PC interface as completion percentages for each assessment criterion. A variety of cases are housed in an online library, and real clinical cases can be uploaded for sharing [6]. How Simodont can serve as a valuable adjunct to traditional preclinical teaching will now be discussed.

## 2. Attributes of Simodont in Preclinical Training

### 2.1. Haptics

Different dental tissues possess different resistance to cutting by the handpiece. Hence, the user experiencing the tactile sensation of drilling into these tissues is important. As dentists may not always be able to see the work area clearly, they sometimes rely on their sense of touch for navigation [7]. With force feedback, the Simodont dental trainer mimics the touch sensation when drilling, preparing cavities, removing caries and delivering other restorative procedures. The tactile feeling is transmitted through the gimbal, and it differs according to each tooth tissue’s hardness level [7]. This improves the resemblance of the cutting resistance of enamel, dentine and pulp, in comparison to the plastic teeth used in conventional simulation. Simodont can also imitate the softness of caries to acquaint students with the feeling of drilling carious lesions [6]. Homogeneous plastic teeth cannot replicate this function. In addition, the trainer detects and records the pressure applied by the trainee’s hand at specific locations [7]. This may help students to gain better control over the force they use and adjust it accordingly to obtain better results.

Typodont teeth are available with different cutting characteristics for enamel, dentine, pulpal tissue and carious tooth structures. These typodont teeth include RTX Caries Teeth (Acadental, Overland Park, KS, USA) and Candent (Candent, Mississauga, ON, Canada). However, no manufacturers have explained how they created typodont teeth that reproduce the cutting characteristics of sound and carious dental tissues. In Simodont, the amount of opposing force that is generated against the handpiece is a function of three determinants [7,8]: (1) the hardness of the virtual material, (2) the drill’s speed and (3) the push force. The hardness of the material is determined according to the grey value of the tooth scan. In addition to issues related to how well the typodont teeth reproduce the cutting characteristics of sound and carious dental tissues, typodont teeth with caries and pulp space are expensive [9,10].

### 2.2. Personnel Cost

As most dental schools do not have adequate dental teachers, generally only a few instructors are available for teaching dental students. The students usually are grouped together and assigned to one teacher, who is responsible for evaluating their work and offering guidance. This teaching model presents a number of challenges. The teacher must provide feedback and address the questions of many students, so students waste time waiting [8]. This reduces the quantity and sometimes quality of the advice they receive. The evaluation system in Simodont provides instantaneous feedback by displaying the completion percentage for each criterion. Students can receive basic evaluation before instructor assessment, which can reduce the time cost. This also alleviates the waiting time for each student. In addition, Simodont allows students to restart if they damage the tooth during a procedure [11] (p. 58). This allows students to be less cautious about making mistakes, especially regarding ‘cutting too much’, which may contribute to faster learning.

### 2.3. Human and Plastic Teeth as Resources

Theoretically, extracted human teeth are the best material for practice. However, the challenges faced to collect them restrict their usage in training. As human teeth supply is unstable, the number of teeth that students can receive is limited and may even be insufficient to carry out proper practice using solely human teeth. Oftentimes, only teeth with extensive damage are extracted, while others have had work performed on them, such as restoration and root canal treatment, which renders them unsuitable for further usage. In addition, occlusal and proximal relations cannot be simulated using extracted human teeth. Furthermore, parents of patients often wish to keep the extracted primary teeth of their children [12].

Plastic models have a standardized size and morphology and an absence of defects. Virtual teeth are produced by scanning extracted teeth with the desired morphology and pathology using cone beam tomography, thus creating a more realistic appearance as compared to plastic teeth [13]. This covers the variation in human dentition more comprehensively. Resources can be pooled using a shared library, as interesting cases can be uploaded for other students to download and work on [11]. This could include cases that are rarely encountered in the clinic, which students otherwise may be unable to see even during their clinical years [6]. Encountering such cases would broaden the range of their experience and improve their understanding of tooth structure and pathology. The translucency of virtual teeth can be adjusted to show the inner structures of the tooth, thus helping students to acquire a more complete understanding of its structure and pathology [6]. The teacher can manipulate and reposition the orientation of the tooth or jaw [6], to simulate a wider range of proximal and occlusal relations. Virtual teeth in Simodont can be regenerated infinitely, which avoids the problem of supply [6]. Students can practice on the same tooth repeatedly until they reach skill competency and familiarity with the morphology, and then choose another tooth to work on. Simodont possesses an online library housing a collection of tooth models, from which a large variety of cases can be downloaded [6]. Virtual teeth usage also skirts the need for disinfection and storage, as well as eliminates the cost of collecting teeth [12].

### 2.4. Diagnosis and Treatment Planning

Proper diagnosis and disease identification must occur before any treatment. While plastic teeth are all standardized and pathology-free, the teeth in Simodont provide a range of healthy and diseased structures for diagnosis and treatment practice. Simodont provides an examination mode and a practice mode, which provides step-by-step guidance for defect and disease recognition [6]. A more detailed examination is possible by magnifying and viewing the model from multiple angles, which has been shown to enhance student understanding of the task at hand and improve students’ confidence in their skills during training [4]. Teachers can add patient records and guiding questions to simulate different clinical situations [6]. Student knowledge is solidified by combining theory and practice. Students can construct their own treatment plans prior to preparation, which can be saved and compared with the actual preparation carried out afterwards for self-assessment.

### 2.5. Instructor Guidance

The dental trainers are connected to a computer, which can display the monitors of six simulators live at once [8]. Teachers then can view the students’ practice through a live screen, without any obstruction from the students’ hands and instruments [6]. Improper techniques and missteps can be spotted by reviewing the procedures being carried out, instead of requiring the instructors to guess the causes of the mistakes just from inspecting the final product. The procedure can also be recorded for future evaluation and comparison [11] (p. 53). The teacher then can provide more accurate corrective advice regarding the performed techniques.

### 2.6. Self-Learning

The Simodont dental trainer displays the completion percentages for each surface as well as over-drilled areas [14]. Continuous feedback is provided throughout the procedure. Students are informed of any mistakes they have made immediately; therefore, they can self-correct on the spot. This can also help to prevent gross over-preparation to the extent that switching to a new virtual tooth is required. The Simodont dental trainer also provides a snapshot function. If a student has difficulty with a certain part of the procedure, they can create a save point, so that they can revisit that particular point in the tooth-preparation procedure [6]. Thus, students can repeat their practice at specific points throughout the preparation sequence, instead of having to start from a new tooth each time. This greatly improves practice effectiveness. The Simodont dental trainer also provides a more flexible learning schedule [15]. Students can use it outside of school hours for review and remediation. This means that students can practice independently at their own pace. This also encourages them to take charge of their own learning.

### 2.7. Manual Dexterity

The Simodont dental trainer provides simple manual dexterity exercises for beginner trainees to develop the basic psychomotor skills needed for restorative work. A virtual block is generated for students to practice drilling, and the requirements of the exercise resemble the principles of cavity preparation [6]. Students then move on to practicing on virtual teeth. Apart from building confidence, students accumulating manual dexterity skills prior to conventional practice prevents them from wasting resources by accidentally causing irreversible damage on plastic teeth [12].

The gimbals on the Simodont dental trainer are designed to resemble the weight and grip of dental hand instruments. A hand rest is also provided for students to practice proper finger grip and hand positioning, which are important skills for students to possess. The position (left or right) of the handpiece gimbal and the mirror gimbal can be exchanged to accommodate people with different dominant sides. A wide range of burs are available for students to practice with [6]. The skills learnt via the dental trainer can be retained and remain transferable to real clinical settings [16]. This suggests that earlier training on a virtual cavity is effective in improving dexterity when students experience a physical model for the first time.

### 2.8. Self-Assessment

The Simodont dental trainer evaluates student work using a set of standard criteria [5]. For a caries-removal exercise, the computer measures the percentages of carious tissue that has been removed and of over-drilled tissue on each side of the cavity. If the amount of over-drilled tissue exceeds a certain margin, immediate failure results. This imitates caries removal in minimally invasive dentistry, in which dentists avoid damaging healthy tissue surrounding a carious lesion. Using the V4 software, the exercise analyses the under- or over-preparedness of the cavity, contour smoothness, depth of the cavity, convergence, divergence, etc., using the Site-Stage (SI/STA) Classification System [17]. This classification system designates the site (SI) and stage (STA) components of the caries lesion and provides guidance on the selection of restorative material. As teachers show the same and standardised criterion for evaluation, students are guided to assess their own outcomes, so that they may gauge the quality of their work and determine key areas for improvement. Students who can actively assess their work will have a better idea about the goal of the treatment simulation they are performing, which may give them clearer direction and bolster their confidence [14].

Self-assessment also allows students to identify potential challenges in learning. By comparing the feedback for different practice runs, students may notice salient performance gaps (for example, the student may score consistently lower for a specific assessment criterion). The students may decide to put more effort into practicing and improving their weaker areas. Variations in the feedback given by different teachers may confuse trainees at an early stage in their career development. Using a standard set of criteria for the machine to generate basic and objective feedback can improve clarity, avoid confusion and instil confidence. Students can learn alternative restorative treatment once they have mastered the basic operative skills. The students’ exercise results are sent to the teaching module. Slower or weaker learners could be identified early and given timely remediation, such that they can catch up with their peers faster.

### 2.9. Other Issues Related to Preclinical Training

The challenge of patient management in paediatrics is that the time for carrying out restorative work is limited. It is important that dentists are able to deliver procedures efficiently, which requires a solid understanding of and familiarity with dental pathology and morphology [12]. Simodont provides pulpotomy and drilling exercises for students to hone their manual dexterity skills on primary dentition. Students can clearly feel the pulp chamber drop using Simodont when they perform simulated root canal treatment. When the students perform crown preparation, they can visualise the original tooth outline on the prepared tooth to aid with comparison and measurement. They also can observe the damage to the neighbouring teeth through the display screen of the Simodont. A summary of the main findings of studies of Simodont in dental education is given in Table 1.

## 3. Limitations of Simodont in Preclinical Training

The haptic limitation of the Simodont dental trainer in tactile sensation in different tooth structures requires improvement. In a pulpotomy exercise, “only 27% of interviewees agreed that the hardness, texture and tactile sensations of Simodont felt realistic for performing pulpotomy and stainless-steel crown exercises” [12]. Furthermore, 79% agreed that conventional simulations felt more realistic. Better simulation fidelity is also required to emulate the texture of pulpal tissues and the mechanical hardness of tooth structures. As mentioned before, another advantage of Simodont is reducing resource wastage. Students do not need to purchase plastic teeth or collect, store or disinfect human teeth for practice. However, the cost of the initial setup and maintenance can be high and will increase the cost of the curriculum. The high cost would ultimately limit the number of devices each individual dental school could afford, as a potential drawback to meeting the training needs of many dental students at one time. Furthermore, students cannot wear loupes while practicing with Simodont due to the need to wear 3D glasses. Many dental schools are training students to perform operative procedures with magnifying loupes, which is becoming an accepted norm amongst dentists and part of the training in simulation laboratory [20]. Additionally, as the trainer only simulates tooth structures, students cannot practice the management of soft tissue such as the tongue and the lips.

## 4. Simodont as an Assessment Tool and Its Future Perspectives

In comparison to traditional entrance examinations involving wire bending or wax carving, an aptitude test conducted using the Simodont dental trainer has a higher resemblance to performing dental restorative work. Candidates’ manual dexterity can be assessed without concerns of injury and the time cost of setting up stations. The potential of the Simodont dental trainer to reduce human bias may prove useful in providing objective assessment for higher-level certification. However, a deeper investigation is warranted into the reliability and evaluation accuracy before this can be implemented [21].

There has been discussion about external accreditation for licensing. This would require (1) a highly reliable means of recording and (2) assessing candidates’ performance according to the criteria set by the examiners. The current challenge lies in acquiring highly reliable data that can prove the candidates’ competence [21]. There is potential for Simodont dental trainer programmes to spread into other dental fields beyond restorative dentistry. Roy et al. [8] discussed the development of a dental hygiene and periodontics programme.

## 5. Conclusions

In conclusion, simulation-based dental education could serve as a powerful aid to preclinical instruction. The Simodont dental trainer provides a platform for students to hone their manual dexterity skills and practice repeatedly prior to conventional preclinical simulation. However, the cost of the initial setup and maintenance can be high. The machine’s force-feedback mechanism provides trainees with the tactile experience of drilling various tissues. It empowers students via self-learning and assessment, with guidance provided for diagnosis and treatment. From training students on basic operative skills to providing basic aptitude tests in entrance examinations, the Simodont dental trainer’s functions and potential for further development may make it a valuable tool for simulation-based dental education.

## Figures and Tables

**Table 1:** Main findings of studies of Simodont in dental education.

Authors, Year [Ref]	Main Findings
De Boer et al., 2016 [18]	Simodont enhanced student performance and appreciation of students working in a virtual learning environment.
Zafer et al., 2020 [12]	Simodont assisted pre-clinical training in restorative procedures in paediatric dentistry.
Murbay et al., 2020 [17]	Simodont facilitated the effectiveness of pre-clinical training in operative dentistry.
Yuan et al., 2021 [19]	Simodont improved the effectiveness in pre-clinical teaching of access and coronal cavity preparation in operative dentistry

## Data Availability

Not applicable.
